# A Flow Cytometry Protocol to Estimate DNA Content in the Yellowtail Tetra *Astyanax altiparanae*

**DOI:** 10.3389/fgene.2017.00131

**Published:** 2017-09-25

**Authors:** Pedro L. P. Xavier, José A. Senhorini, Matheus Pereira-Santos, Takafumi Fujimoto, Eduardo Shimoda, Luciano A. Silva, Silvio A. dos Santos, George S. Yasui

**Affiliations:** ^1^National Center for Research and Conservation of Continental Fish, Chico Mendes Institute of Biodiversity Conservation Pirassununga, Brazil; ^2^Aquaculture Center, Sao Paulo State University Jaboticabal, Brazil; ^3^Faculty of Fisheries Sciences, Hokkaido University Hakodate, Japan; ^4^Department of Pharmacy, Cândido Mendes University Rio de Janeiro, Brazil; ^5^Department of Veterinary Medicine, University of Sao Paulo Pirassununga, Brazil; ^6^AES Tietê Promissão, Brazil

**Keywords:** yellowtail tetra, ploidy status, fish, flow cytometry, sample preservation

## Abstract

The production of triploid yellowtail tetra *Astyanax altiparanae* is a key factor to obtain permanently sterile individuals by chromosome set manipulation. Flow cytometric analysis is the main tool for confirmation of the resultant triploids individuals, but very few protocols are specific for *A. altiparanae* species. The current study has developed a protocol to estimate DNA content in this species. Furthermore, a protocol for long-term storage of dorsal fins used for flow cytometry analysis was established. The combination of five solutions with three detergents (Nonidet P-40 Substitute, Tween 20, and Triton X-100) at 0.1, 0.2, and 0.4% concentration was evaluated. Using the best solution from this first experiment, the addition of trypsin (0.125, 0.25, and 0.5%) and sucrose (74 mM) and the effects of increased concentrations of the detergents at 0.6 and 1.2% concentration were also evaluated. After adjustment of the protocol for flow cytometry, preservation of somatic tissue or isolated nuclei was also evaluated by freezing (at −20°C) and fixation in saturated NaCl solution, acetic methanol (1:3), ethanol, and formalin at 10% for 30 or 60 days of storage at 25°C. Flow cytometry analysis in yellowtail tetra species was optimized using the following conditions: lysis solution: 9.53 mM MgCl_2_.7H_2_0; 47.67 mM KCl; 15 mM Tris; 74 mM sucrose, 0.6% Triton X-100, pH 8.0; staining solution: Dulbecco's PBS with DAPI 1 μg mL^−1^; preservation procedure: somatic cells (dorsal fin samples) frozen at −20°C. Using this protocol, samples may be stored up to 60 days with good accuracy for flow cytometry analysis.

## Introduction

The yellowtail tetra, *Astyanax altiparanae*, has been highlighted as a great model fish for laboratory studies, aquaculture (Prioli et al., 2002; dos Santos et al., 2016), basics and applied research for reproductive technologies, conservation of valuable genetic resources and establishment of gene banks for endangered species using surrogate technologies (Yamaha et al., 2007).

Surrogate propagation permits a fish to produce gametes from another species, and in this case, the yellowtail tetra is strategic because this species can be manipulated to produce gametes from related endangered species. This technology requires sterile host individuals to ensure the production of exogenous gametes. Some protocols established the sterilization of the yellowtail tetra by chronic exposure to high temperatures. However, sterilization was maintained only for a few weeks (de Siqueira-Silva et al., 2015). On the other hand, artificial induction of triploid by chromosome set manipulation using temperature shock has been promising in several fish species like tilapia and yellowtail tetra may be an efficient method to obtain permanent sterile individuals (Mair, 1993; Piferrer et al., 2009). In previous work (Adamov et al., 2016; do Nascimento et al., 2017), a protocol was established to produce triploid yellowtail tetra, and the ploidy status was confirmed by flow cytometry using a general protocol for plants and other animal species, but several samples could not be analyzed due to inaccurate analysis. It suggests that there is a specific protocol needed for this fish species.

Identification of triploids can be achieved using different methods including nuclear volume of fish erythrocytes (Allen and Stanley, 1978, 1979) and chromosome counts by karyotype (Allen et al., 1982). However, flow cytometry analysis is currently the most effective, rapid, and accurate method to identify the ploidy status. Flow cytometry analysis is a valuable tool for chromosomal studies in fish species, including cell cycle analysis, confirmation of the ploidy status, and determination of genome size in different fish species including the loach (Zhou et al., 2008), Atlantic salmon (Allen, 1983), *Poecilia formosa* (Lampert et al., 2008), and other teleost species (Ciudad et al., 2002; Zhu et al., 2012). However, the most protocols used for those analysis employs especially propidium iodide protocols and commercial kits that despite exhibit successfully results are usually expensive and not tested for fish species.

In such analysis, tissue sampling is critical since samples rapidly loss the viability if they are fresh and quality if previous fixatives was used for flow cytometry analysis. However, some times such samples are usually obtained in field conditions and have to be transported alive into the laboratory facilities for subsequent analysis. Furthermore, it is usually necessary to process large number of samples and such a situation require the development of protocols for long-term storage of these samples during the analysis. Some protocols are available for species such as the grass carp (Brown et al., 2000), Chinese grass carp (Burns et al., 1986), Atlantic salmon and shellfish (Allen, 1983), but there is not a protocol for Neotropical species like the yellowtail tetra.

Therefore, the aim of this study was firstly to establish a two-step protocol to estimate the DNA content in the yellowtail tetra, *A. altiparanae* by flow cytometry. In addition, an efficient protocol for long-term storage of tissue used for subsequent flow cytometry analysis was established. The combination of both procedures may give rise to a reliable determination of ploidy status in the yellowtail tetra and other important related species.

## Materials and methods

All the procedures were accepted by the Guide for the Care and Use of Laboratory Animals of CEPTA (CEUA #02031.000033/2015-11).

### General procedure for flow cytometry

Adult yellowtail tetra *A. altiparanae* were collected from the Mogi-Guassu river, São Paulo State, Brazil (21.925706 S, 47.369496 W). Males and females were used for the analysis, but no differences in DNA content between sexes were found for this species (Martinez et al., 2012). A small piece of dorsal fin (~2 mm^2^) was clipped from the fish and then placed in a 1.5 mL microtube containing 120 μL of lysing solution. This lysing solution was prepared using detergents and other components (see following experiment sections). The samples were incubated at room temperature for 30 min with occasional mixing. Staining was achieved by addition 800 μL of Calcium-Free Dulbecco's PBS (Sigma #D5773) containing DAPI at 1 μg mL^−1^. The samples were then filtered by a 30-μm mesh (Celltrics, Partec GmBH, Germany). Stained samples were then analyzed by a Partec CyFlow Plody Analyzer (Partec GmBH, Germany) with a specific filter set for DAPI excitation (358 nm). As a control group, a commercial kit specific for aquaculture and plants (Partec CyStain DNA 2-step, Partec GmBH, Germany) was used.

### Solutions and detergents for cell lysing

In this study, five (1–5) solutions (S) for cell lysing based on plant flow cytometry were compared: **S1** (Dpoolezel et al., 1989): 15 mM Tris, 2 mM Na_2_EDTA, 80 mM KCl, 20 mM NaCl; **S2** (Arumuganathan and Earle, 1991): 9.53 mM MgCl_4_, 47.67 mM KCl, 15 mM Tris; **S3** (Galbraith et al., 1983): 45 mM MgCl_2_, 30 mM sodium citrate, 2 mM NaHCO_3_; **S4** (Marie and Brown, 1993): 50 mM glucose, 15 mM KCl, 15 mM NaCl, 5 mM Na_2_EDTA, 50 mM sodium citrate; **S5** (Pfosser et al., 1995): 200 mM tris, 4 mM MgCl_2_.6H_2_O. In each solution mentioned above, three detergents (Tween-20, triton-X, and nonidet P-40 substitute) were added at three concentrations each: 0.1, 0.2, and 0.4%. The combination of five solutions, three detergents at three concentrations, and the control group (commercial kit) gave rise to 46 lysing treatments. Dorsal fin samples were processed in each solution as mentioned above and then were checked for the best results according to the peak quality. This experiment was performed in triplicates.

### Evaluation of high concentrations of detergents, trypsin, and sucrose additions

Using the best combination of detergents and the solution obtained above (i.e., Arumuganathan and Earle, 1991), detergent concentrations at 0.15, 0.3, 0.6, and 1.2% were evaluated with or without the addition of sucrose (74 mM). Using the best result, the effects of trypsin addition (at 0.125, 0.25, and 0.5%) were also evaluated on peak quality.

### Fixatives and preservation strategies

In this experiment, two kinds of samples (fish fin or isolated nuclei) were evaluated. For each of them, five procedures were evaluated: freezing at −20°C, ethanol 70%, acetic methanol (1 part of acetic acid:3 parts of methanol), saline solution (saturated NaCl solution), and formalin at 10%. For preservation of tissue, about ~2 mm^2^ of dorsal fin from the *A. altiparanae* were placed in 1.5 mL microtubes containing 1 mL of each of fixatives and maintained at 25°C in a BOD incubator under dark conditions. For freezing, the sample was placed in a 1.5 mL microtube containing 200 μL of 0.9% NaCl and then directly frozen at −20°C in a biomedical freezer. Isolated nuclei were obtained from dorsal fins (~2 mm^2^) using 100 μL of lysing solution obtained in previous experiment. For preservation of nuclei, 1 mL of each fixative was added to each nuclei suspension and then maintained at 25°C. Nuclei suspensions were also frozen at −20°C. Flow cytometry analysis were performed at 30 and 60 days of storage. Fixed tissue was collected and washed in Dulbecco's PBS and then processed in lysing solution before staining. Nuclei suspensions were centrifuged at 12,000 G for 2 min, and the fixatives were removed by pipetting. The nuclei pellet was re-suspended in staining solution and then analyzed on flow cytometer. As control groups, fresh (non-fixed) samples from day 0, 30, and 60 were used. For each of the 21 treatments, 10 replications were used.

### Statistical analysis

Data are shown as mean ± SD. Data were checked for normality using the Liliefor test and also compared using ANOVA followed by Tukey's multiple range test (*P* < 0.05). Statistica version 11 were used for statistical analysis.

## Results

### Solutions and detergents for cell lysing

**Figure 1:**
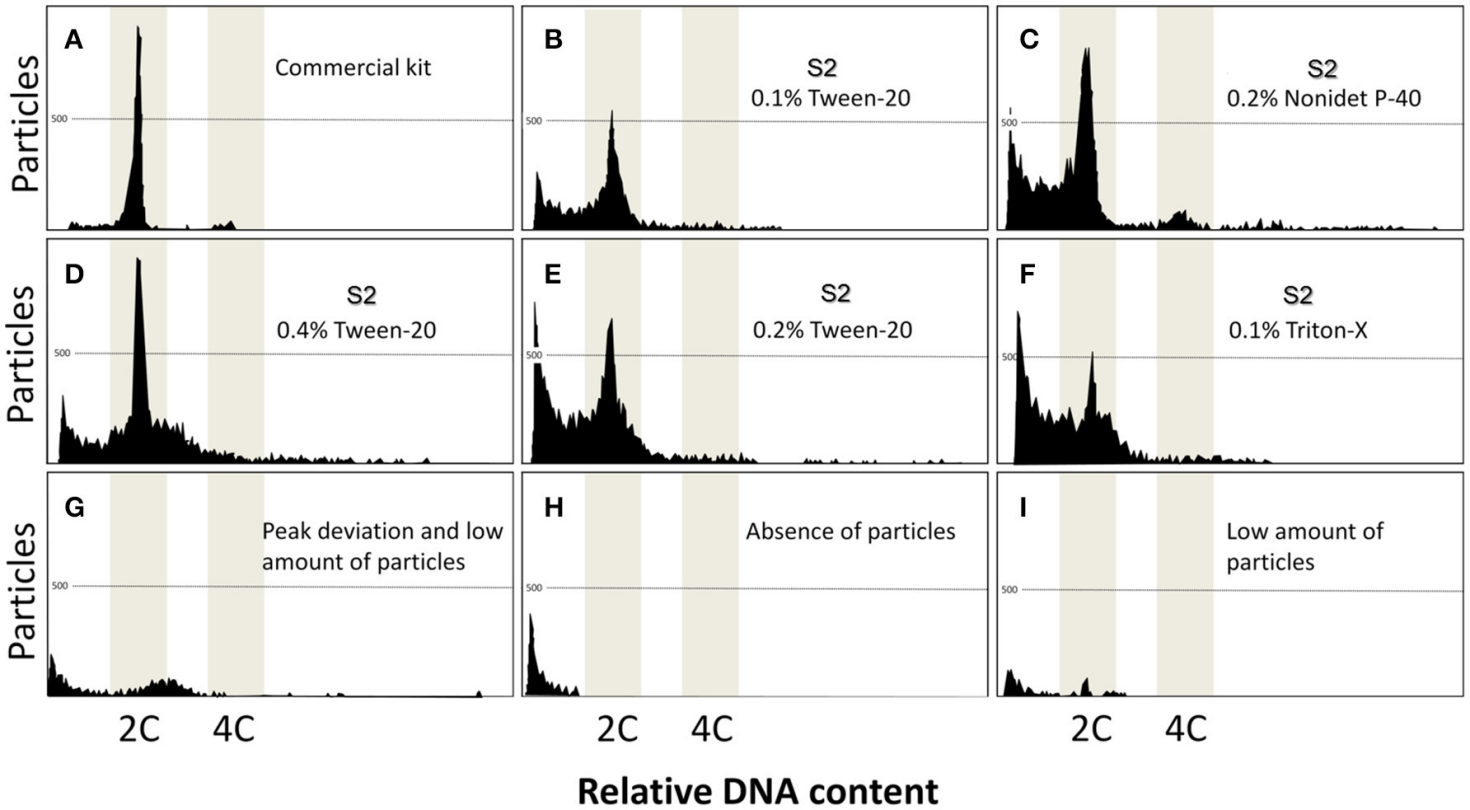
Flow cytometric charts of fin samples using various lysing treatments and staining with DAPI. A combination of five solutions, and three detergents (Triton-X, Tween-20, and Nonidet *P*-40 at 0.1, 0.2, and 0.4%) gave rise to 45 treatments. Additionally, a commercial kit was used as control group. Commercial kit **(A)** produced the clear peaks. S2 associated with Tween-20 **(B,D,E)**, Nonidet **(C)** and Triton-X **(F)** gave some unclear peaks, demonstrating some potencial for analysis. Other treatments produced poor results including peak deviation **(G)**, absence of particles **(H)**, and low amount of particles **(I)**.

In the control group that used commercial kit, two clear peaks arose: one with 2C content and the other with 4C, originating from dividing cells (Figure 1A). On the other hand, none of the combinations of the solutions and detergents evaluated generated clear peaks. Problems included noisy peaks (Figures 1B–F), deviation in the DNA content (Figure 1G), absence of peaks (Figure 1H), and in most cases, low amounts of particles were found (Figure 1I). As observed in Table 1, the only solution that kept the same relative DNA content in comparison to the control, independent of the detergent or concentration, was the S2. Furthermore, the S2 solution with Triton at 0.1% presented a good number of particles isolated and the best peak quality in terms of the histograms (Table 1). All other solutions presented poor results in regards to quality of peaks, DNA content, and number of particles. Considering the quality of peaks, DNA content, and the number of particles, S2 presented the best results and was used in later experiments.

**Table 1:** Evaluation of solutions and detergent concentrations for flow cytometry in fish.

**Solution**	**Detergent**	**Concentration (%)**	**Relative DNA content**	**Number of Particles**
Commercial kit (Control)	Unknown	Unknown	1.0 ± 0.0^a^	9058.3 ± 9238.0^a^
S1	Nonidet P-40	0.1	0.7 ± 0.6^a^	217.0 ± 271.2^abc^
		0.2	1.1 ± 0.0^a^	451.7 ± 254.5^abc^
		0.4	0.0 ± 0.0^b^	0.0 ± 0.0^c^
	Triton-X	0.1	0.7 ± 0.6^a^	114.3 ± 125.1^abc^
		0.2	0.7 ± 0.6^a^	275.3 ± 396.7^abc^
		0.4	1.0 ± 0.0^a^	43.3 ± 27.3^bc^
	Tween 20	0.1	0.0 ± 0.0^b^	0.0 ± 0.0^c^
		0.2	0.0 ± 0.0^b^	0.0 ± 0.0^c^
		0.4	0.7 ± 0.6^a^	116.7 ± 108.3^abc^
S2	Nonidet P-40	0.1	1.0 ± 0.0^a^	235.0 ± 193.0^abc^
		0.2	1.0 ± 0.0^a^	980.0 ± 1249.7^abc^
		0.4	1.0 ± 0.0^a^	565.3 ± 298.1^abc^
	Triton-X	0.1	1.0 ± 0.0^a^	1342.3 ± 261.2^abc^
		0.2	1.0 ± 0.0^a^	389.0 ± 374.2^abc^
		0.4	1.0 ± 0.0^a^	43.7 ± 23.4^bc^
	Tween 20	0.1	1.0 ± 0.0^a^	820.3 ± 542.3^abc^
		0.2	1.0 ± 0.0^a^	1249.3 ± 978.6^abc^
		0.4	1.0 ± 0.0^a^	1555.0 ± 604.6^abc^
S3	Nonidet P-40	0.1	1.0 ± 0.0^a^	205.0 ± 120.2^abc^
		0.2	1.0 ± 0.0^a^	222.7 ± 107.6^abc^
		0.4	0.9 ± 0.0^a^	652.0 ± 275.0^abc^
	Triton-X	0.1	0.3 ± 0.6^a^	125.3 ± 217.1^abc^
		0.2	0.7 ± 0.6^a^	127.3 ± 145.6^abc^
		0.4	0.0 ± 0.0^b^	0.0 ± 0.0^c^
	Tween 20	0.1	1.0 ± 0.0^a^	1501.3 ± 827.4^ab^
		0.2	1.0 ± 0.0^a^	203.7 ± 203.0^abc^
		0.4	0.3 ± 0.6^a^	317.3 ± 148.1^abc^
S4	Nonidet P-40	0.1	0.4 ± 0.6^a^	155.3 ± 269.1^abc^
		0.2	1.1 ± 0.0^a^	594.7 ± 53.5^abc^
		0.4	1.0 ± 0.0^a^	820.3 ± 931.3^abc^
	Triton-X	0.1	0.7 ± 0.6^a^	176.7 ± 172.2^abc^
		0.2	0.7 ± 0.6^a^	538.0 ± 589.4^abc^
		0.4	0.7 ± 0.6^a^	596.3 ± 525.4^abc^
	Tween 20	0.1	0.3 ± 0.6^a^	294.3 ± 509.8^ab^
		0.2	0.7 ± 0.6^a^	741.3 ± 657.5^abc^
		0.4	1.0 ± 0.0^a^	352.7 ± 128.7^abc^
S5	Nonidet P-40	0.1	1.1 ± 0.0^a^	220.0 ± 86.3^abc^
		0.2	1.0 ± 0.0^a^	295.7 ± 213.3^abc^
		0.4	1.0 ± 0.0^a^	327.0 ± 350.9^abc^
	Triton-X	0.1	1.0 ± 0.0^a^	201.0 ± 170.6^abc^
		0.2	1.1 ± 0.0^a^	161.3 ± 78.1^abc^
		0.4	1.0 ± 0.0^a^	130.7 ± 154.7^abc^
	Tween 20	0.1	1.1 ± 0.0^a^	671.3 ± 469.8^abc^
		0.2	0.7 ± 0.6^a^	959.0 ± 1239.8^abc^
		0.4	1.0 ± 0.0^a^	590.7 ± 481.8^abc^

*Pieces of fin (~2 mm^2^) were treated with the solutions and detergents combinations below, stained with DAPI, and analyzed by flow cytometry. Different letters within the columns indicates significant differences by Tukey test (P < 0.05)*.

### Evaluation of high concentrations of detergents, trypsin and sucrose additions

**Figure 2:**
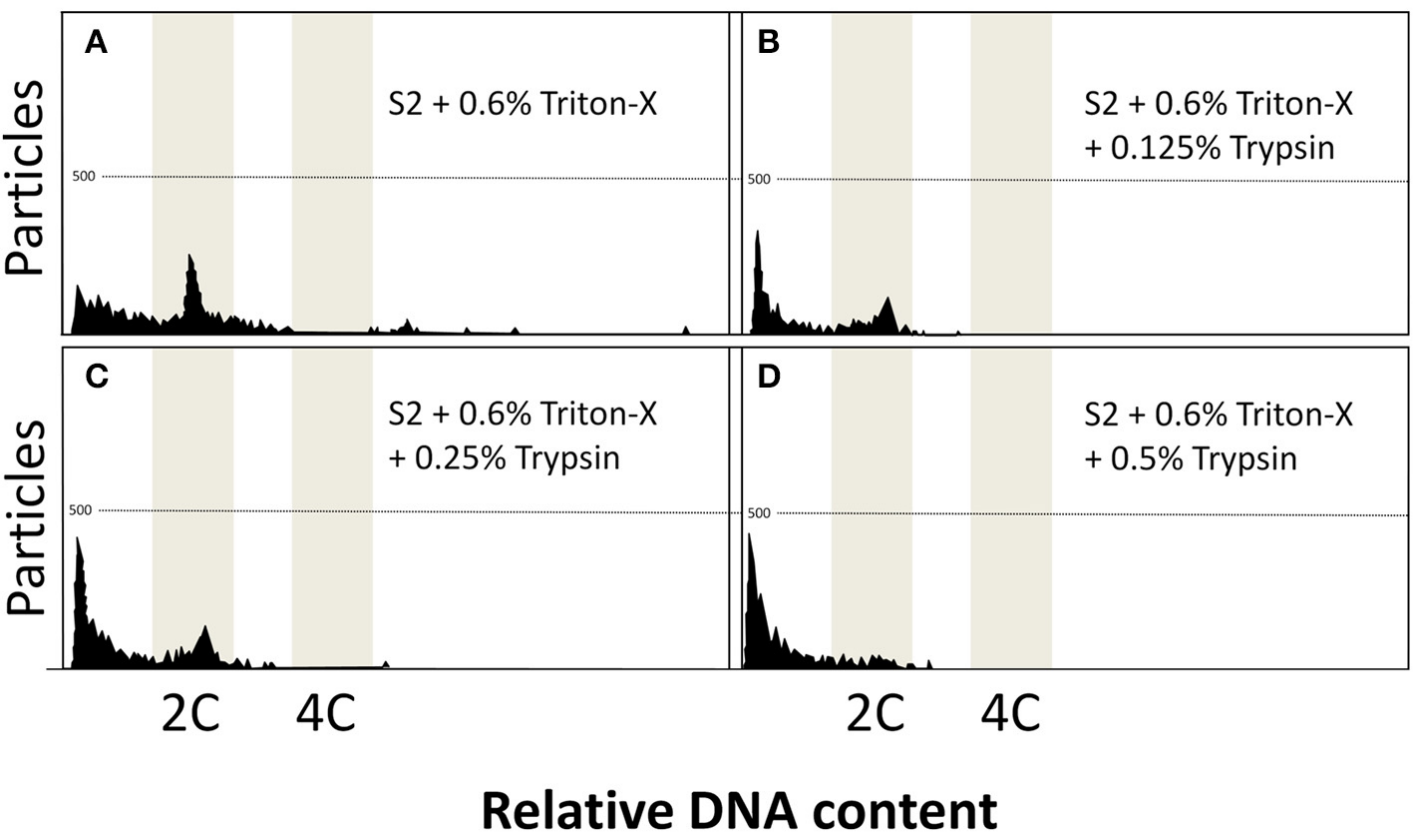
Flow cytometric charts of fin samples using various lysing treatments and staining with DAPI. Solution S2 was combined with three detergents (Triton-X, Tween-20, and Nonidet P-40 at 0.15, 0.3, 0.6, and 1.2%). Trypsin additions at 0.125, 0.25, and 0.5% was also evaluated. Triton-X at 0.6% gave some noisy peaks **(A)**. Trypsin additions produced reduced peaks **(B–D)**.

As observed in Figure 2, the addition of trypsin at 0.125 (Figure 2B), 0.25 (Figure 2C), and 0.5% (Figure 2D) presented detrimental effects on the flow cytometry analysis, and lower peaks arose with increasing concentrations of trypsin. Such data coincided with lower amounts of particles (Table 2), although it was significantly decreased only at 0.5% (74.3 ± 24.0, *P* = 0.0491). The DNA content did not present any statistical differences (*P* = 0.0578) and ranged from 0.99 ± 0.03 (at 0.5% trypsin) to 1.04 ± 0.01 (at 0.125% trypsin).

**Table 2:** Evaluation of trypsin additions for flow cytometry in fish.

**Solution**	**Detergent**	**Concentration (%)**	**Trypsin addition (%)**	**Relative DNA content**	**Number of Particles**
S2 + 74 mM sucrose	Triton-X	0.6	0	1.00 ± 0.01^a^	715.0 ± 318.2^a^
			0.125	1.04 ± 0.01^a^	400.7 ± 358.5^ab^
			0.25	1.03 ± 0.02^a^	139.7 ± 86.6^ab^
			0.5	0.99 ± 0.03^a^	74.3 ± 24.0^b^

*S2 with 0.6% Triton-X was combined with trypsin at 0.125, 0.25, and 0.5% and 74 mM Sucrose and measured the relative DNA content and number of isolated particles. Pieces of fin (2 mm^2^) were treated with the solutions, stained with DAPI and analyzed by flow cytometry. Different letters within the columns indicates significant differences by Tukey test (P < 0.05)*.

**Figure 3:**
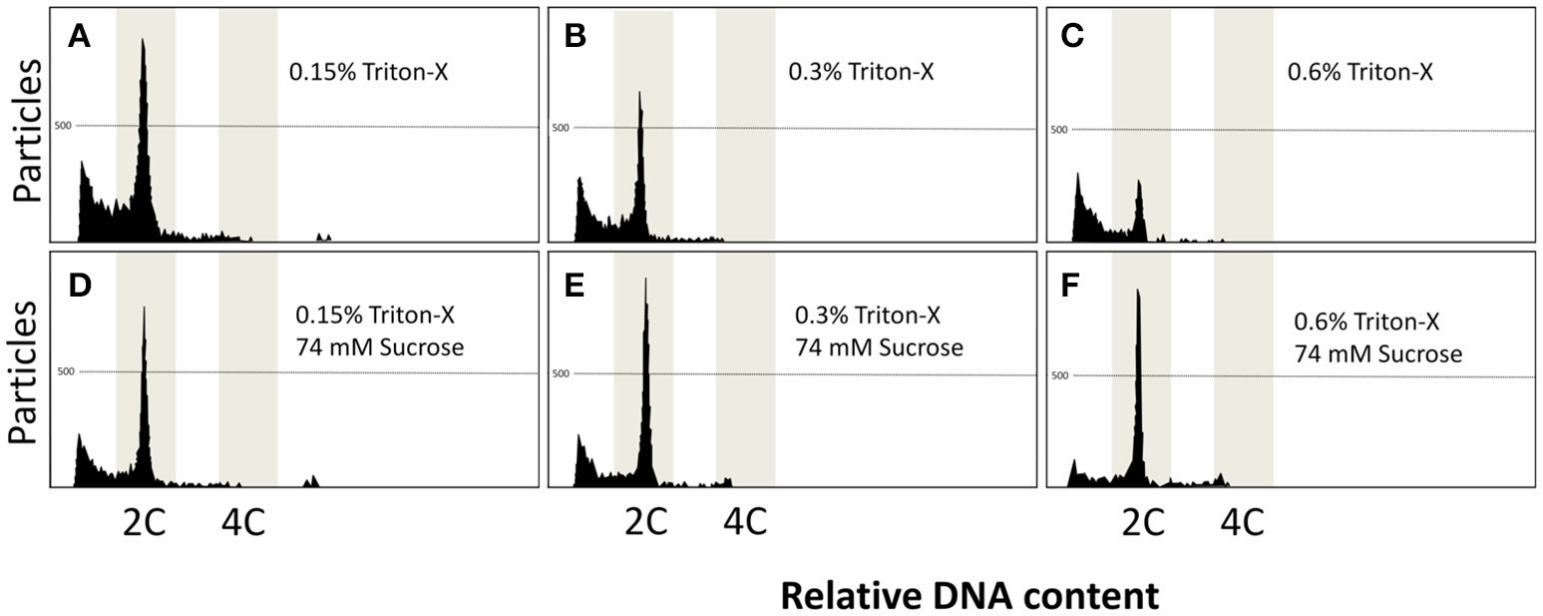
**(A–F)** Flow cytometric charts of fin samples using various lysing treatments and staining with DAPI. A combination of Triton-X 0.15, 0.3, and 0.6% was associated with sucrose at 74 mM. Triton-X produced noisy peaks at all concentrations. The addition of sucrose improved the results, giving rise to clear peaks.

Flow cytometry using S2 solution associated with triton-X at 0.15, 0.3, and 0.6% gave rise to noisy peaks (Figures 3A–C). The addition of sucrose at 74 mM associated with triton-X at the same concentrations produced clearer peaks, and at 0.3% (Figure 3E) and 0.6% (Figure 3F), but not at 0.15% (Figure 3D), two clear 2 and 4C peaks arose. Relative DNA content (Table 3) was decreased in Triton-X at 1.2% (0.85 ± 0.03 C, *P* = 0.0155), and sucrose with Triton-X at 0.15% (0.87 ± 0.02 C, *P* = 0.0492), 0.3% (0.85 ± 0.01 C, *P* = 0.0155), and 0.6% (0.84 ± 0.02 C; *P* = 0.1054). The number of particles from treatment S2 with triton-X at 0.125% was 927.0 ± 132.4 and did not present statistical differences (*P* = 0.0538) when compared with the control (2,716.3 ± 1,822.2). However, other treatments had a significant decrease and ranged from 194.7 ± 37.1 (S2 and triton-X at 0.6%) to 826.3 ± 67.0 (S2 with 0.3% triton X and 74 mM sucrose).

**Table 3:** Evaluation of sucrose addition for flow cytometry in fish.

**Solution**	**Detergent**	**Concentration (%)**	**Relative DNA content**	**Number of Particles**
Commercial kit (Control)	Unknown	Unknown	1.00 ± 0.08^a^	2716.3 ± 1822.2^a^
S2	Triton-X	0.15	0.92 ± 0.04^ab^	927.0 ± 132.4^ab^
		0.30	0.88 ± 0.02^ab^	343.3 ± 209.2^b^
		0.60	0.88 ± 0.04^ab^	194.7 ± 37.1^b^
		1.20	0.85 ± 0.03^c^	176.0 ± 257.3^b^
S2 + 74 mM Sucrose	Triton-X	0.15	0.87 ± 0.02^c^	369.3 ± 337.8^b^
		0.30	0.85 ± 0.01^c^	826.3 ± 67.0^b^
		0.60	0.84 ± 0.02^c^	776.3 ± 100.2^b^
		1.20	0.93 ± 0.09^ab^	341.7 ± 139.5^b^

*S2 was associated Triton-X at 0.15, 0.30, 0.60, 1.20% and sucrose at 74 mM and measured the DNA content and number of particles. Pieces of fin (2 mm^2^) were treated with the solutions, stained with DAPI and analyzed by flow cytometry. Different letters within the columns indicates significant differences by Tukey test (P < 0.05)*.

**Figure 4:**
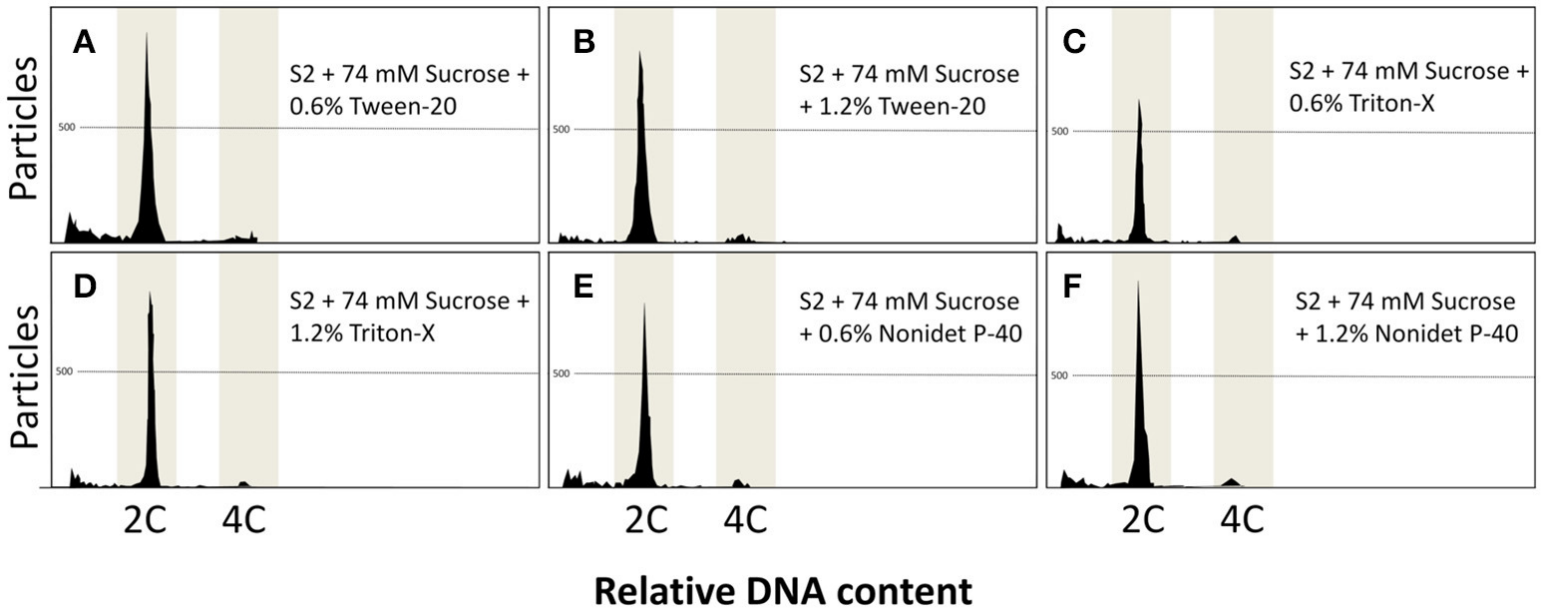
**(A–F)** Flow cytometric charts of fin samples using various lysing treatments and staining with DAPI. A combination of solution S2 with Triton-X, Tween-20, and Nonidet P-40 at 0.6 and 1.2% and 74 mM sucrose was evaluated. All treatments gave rise to clear peaks.

As there were promising results with 0.6% triton-X, higher concentrations of other detergents at 0.6 and 1.2% were also evaluated. The results using S2 and sucrose presented good results and Triton-X, Tween-20, and Nonidet P-40 presented similar results, both at 0.6 and 1.2% (Figure 4), with a main peak at 2C and a secondary peak at 4C. The DNA content did not present significant differences (*P* = 0.0779) within all the detergents and concentrations evaluated in this study and ranged from 0.99 ± 0.01 C (1.2% Nonidet P-40) to 1.03 ± 0.01 C (1.2% Tween-20). Similarly, all the treatments did not present significant differences regarding the number of particles (*P* = 0.0602), and this parameter ranged from 725.3 ± 119.0 (0.6% triton) to 1095.3 ± 183.4 (1.2% Tween-20).

Based on the results above, Triton-X at 0.6% gave better results on nuclei isolation (Table 4) and subsequent flow cytometric analysis (Figure 4C) and, thus, was then chosen for later experiments.

**Table 4:** Evaluation of S2 solution associated with high concentrations of detergents (0.6 and 1.2%) regarding the relative DNA content and number of isolated particles.

**Solution**	**Detergent**	**Concentration (%)**	**Relative DNA content**	**Number of particles**
S2	Nonidet P-40	0.6	0.99 ± 0.01^a^	829.0 ± 45.9^a^
		1.2	0.99 ± 0.01^aa^	763.0 ± 175.3^a^
	Triton-X	0.6	1.02 ± 0.02^a^	725.3 ± 119.0^a^
		1.2	0.99 ± 0.03^a^	805.3 ± 189.6^a^
	Tween 20	0.6	1.01 ± 0.01^a^	1090.7 ± 228.7^a^
		1.2	1.03 ± 0.01^ac^	1095.3 ± 183.4^a^

*Pieces of fin (2 mm^2^) were treated with the solutions, stained with DAPI, and analyzed by flow cytometry. Different letters within the columns indicates significant differences by Tukey test (P < 0.05)*.

### Fixatives and preservation strategies

**Figure 5:**
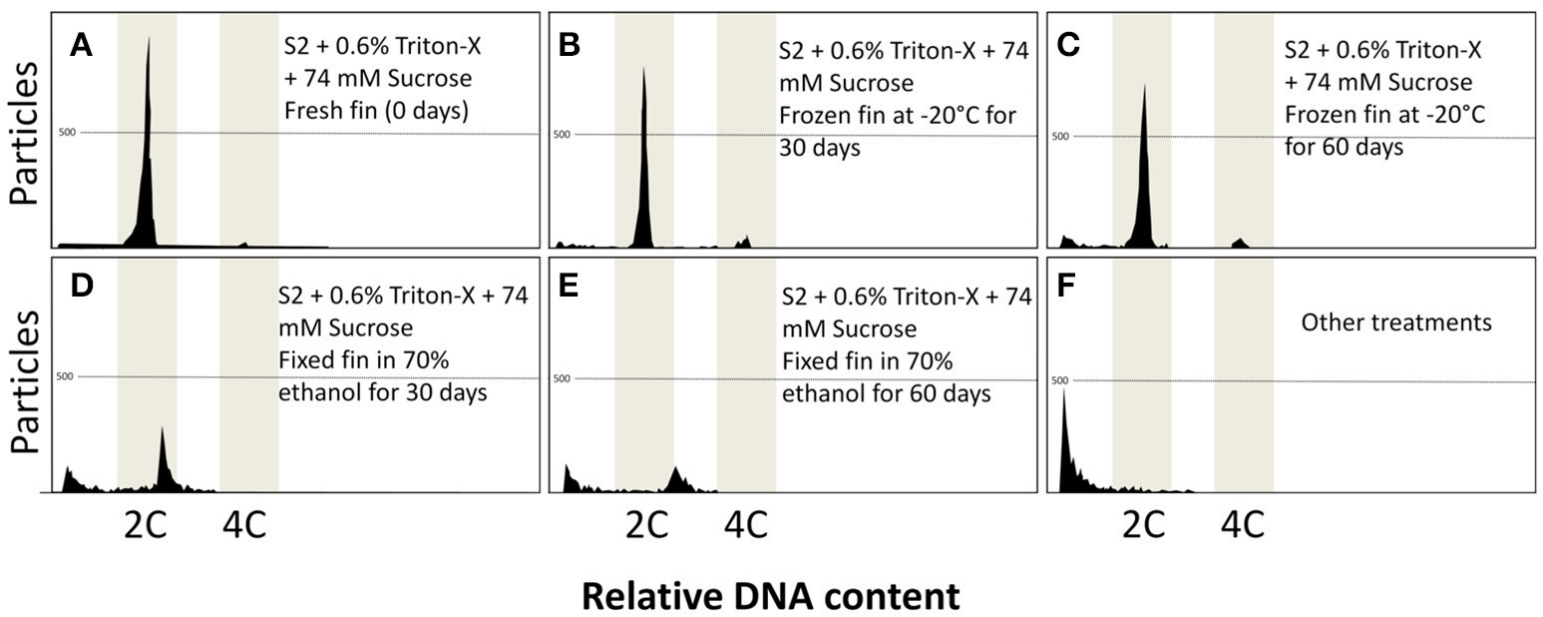
Flow cytometric charts of fin samples and isolated nuclei preserved using various procedures at 0, 30, and 60 days of storage. Frozen fins at −20°C gave the best results and storage at 30 days **(B)** and 60 days **(C)** did not differ from fresh samples (0 days) **(A)**. Fixation of fin in 70% ethanol gave peaks with low amount of particles and peak deviation at 30 days **(D)** and 60 days **(E)**. Other treatments did not generate any data **(F)**.

After 30 and 60 days of fixation, only frozen dorsal fin samples maintained the ability for flow cytometric analysis (Figure 5). Very clear peaks arose (Figures 5B,C), like samples from the control groups with fresh tissue (Figure 5A). Preserved dorsal fin using 70% ethanol produced peaks after 30 and 60 days (Figures 5D,E), but deviation of the peak occurred in both cases. Other fixatives gave rise to noisy peaks or, in most cases, did not show any peak (Figure 5F). The preservation of isolated nuclei as a new strategy failed in all cases giving rise to noisy or very few particles.

After preservation in 70% ethanol, fin samples presented increased DNA content in flow cytometry analysis after 30 days (1.14 ± 0.04; *P* = 0.0001) and 60 days (1.21 ± 0.06 C; *P* = 0.0001) when compared with control group (1.00 ± 0.02 C) (Table 5). Nucleus preserved in formaldehyde after 30 days presented decreased DNA content (0.86 ± 0.04; *P* = 0.0001). A similar result was observed for fin samples preserved with formaldehyde for 60 days (0.80 ± 0.07; *P* = 0.0001) and nucleus preserved for 60 days with salt (0.89 ± 0.10 C; *P* = 0.0011), formaldehyde (0.78 ± 0.04 C; *P* = 0.0001), ethanol (0.72 ± 0.03 C; *P* = 0.0001), and methanol (0.72 ± 0.05 C; *P* = 0.0001).

**Table 5:** Preservations strategies (Salt, Formaldehyde, Ethanol, Methanol, and Freeze) for flow cytometry in fish.

**Storage**	**Sample**	**Treatment**	**RelativeDNA content**	**Number of particles**
0 days	Fin	Control	1.00 ± 0.02^cde^	2215.60 ± 1015.94^a^
30 days	Fin	Salt	0.98 ± 0.10^def^	13.80 ± 17.27^c^
		Formaldehyde	0.94 ± 0.03^efgh^	32.00 ± 21.58^c^
		Ethanol	1.14 ± 0.04^ab^	13.30 ± 18.96^c^
		Methanol	1.07 ± 0.02^bc^	8.10 ± 8.32^c^
		Freeze	1.01 ± 0.02^cde^	892.90 ± 357.95^b^
	Nucleus	Salt	0.99 ± 0.06^cdef^	8.90 ± 11.11^c^
		Formaldehyde	0.86 ± 0.04^hi^	48.20 ± 24.50^c^
		Ethanol	1.03 ± 0.03^cd^	102.80 ± 27.47^c^
		Methanol	0.94 ± 0.05^efgh^	222.90 ± 207.72^c^
		Freeze	0.99 ± 0.05^cdef^	42.90 ± 60.24^c^
0 days	Fin	Control		
60 days	Fin	Salt	0.96 ± 0.03^defg^	0.60 ± 0.70^c^
		Formaldehyde	0.80 ± 0.07^ij^	15.30 ± 9.45^c^
		Ethanol	1.21 ± 0.06^a^	25.70 ± 28.86^c^
		Methanol	0.98 ± 0.08^def^	4.90 ± 8.84^c^
		Freeze	0.98 ± 0.02^def^	862.20 ± 467.83^b^
	Nucleus	Salt	0.89 ± 0.10^gh^	3.50 ± 4.33^c^
		Formaldehyde	0.78 ± 0.04^ij^	36.90 ± 15.15^c^
		Ethanol	0.72 ± 0.03^j^	146.20 ± 44.50^c^
		Methanol	0.72 ± 0.05^j^	21.20 ± 10.67^c^
		Freeze	0.91 ± 0.06^fgh^	5.40 ± 4.17^c^

*Pieces of fin (2 mm^2^) and isolated nuclei were fixed with different procedures. The samples were analyzed at 0 (control), 30 and 60 days by flow cytometry and obtained the relative DNA content and number of isolated particles. Different letters within the columns indicates significant differences by Tukey test (P < 0.05)*.

The number of particles was reduced in all cases when compared with fresh samples (2215.6 ± 1015.9; *P* = 0.0001). However, the best results were achieved with frozen dorsal fins after 30 days (892.9 ± 357.9) and 60 days (862.2 ± 467.8).

## Discussion

Successful flow cytometry analysis depends on several aspects including the interaction of the sample and the components of lysing solutions (Loureiro et al., 2007). An adequate combination of osmolality, buffers, stabilizers, and detergents is then required to achieve good results. A two-step procedure with cell lysing and nuclear staining, solubilization of cell membrane and subsequent preservation of nuclear envelope is the key point for flow cytometry, and combination of detergents, solutions, and membrane stabilizer was successful for the studied species. The current results indicates that solutions containing magnesium, presented better results during flow cytometry analysis. This is assumed to be the case because of magnesium ions work as a cromatin stabilizer (Galbraith et al., 1983) and then we conclude that this component is important in order to preserve nuclear membrane of the yellowtail tetra. Similarly, sucrose additions presented good results. Sucrose affects osmolality and presents membrane stabilizer ability and is also widely used in several process including cell lysis and cryopreservation ir order to maintain the membrane characteristics (Medina-Robles et al., 2005). Furthermore, sucrose is important for the maintenance of nuclear integrity (Marie and Brown, 1993). Non-ionic detergents used in this study efficiently solubilized cell membrane, but the nuclei were kept intact, indicating that lysing and stabilizing process is membrane-specific.

In this work, a protocol for sample storage and subsequent flow cytometry analysis was achieved. Such a procedure is applicable for long-term preservation for future analysis. The preservation of marine bacteria under fixation with paraformaldehyde and storage in liquid nitrogen reduced the cell count and generated multiple peaks in flow cytometry analysis (Kamiya et al., 2007). Similar decrease in quality was observed in marine bacteria and algae (Troussellier et al., 1993). In this study, when sub-zero temperatures at −20° C were used the peak quality and cell concentration was maintained for 60 days of storage providing the good results in flow cytometry. Such a new procedure for preservation of fish samples may be used in many fields including aquaculture and biomedicine.

A new procedure for fixation of samples for flow cytometry was evaluated, including isolated nuclei instead of tissue, however, further work is required to improve the method, results as well as the alternative comparisons of different fixatives and ways of storage. However, such an approach of preserving an isolated nucleus may be interesting because fixation and storage of tissue may affect membrane characteristics (Suganuma and Morioka, 1979). It can also reduce the efficiency of lysing and staining procedures, and, thus, the nucleus cannot be isolated in some cases. Nuclear isolation and preservation may be applicable as a preservation procedure for flow cytometry analysis.

In conclusion, a simple and inexpensive protocol for DNA content analysis by flow cytometry in yellowtail tetra *A. altiparanae* and sample preservation was established, as follows: lysing solution for fish samples composed by 9.53 mM MgCl_2_.7H_2_O, 47.67 mM KCl, 15 mM Tris, 74 mM sucrose, and 0.6% Triton-X. Tissue samples of fish may be preserved at −20°C for 60 days for future analysis using such a protocol.

## Author contributions

PX: Acquisition, analysis, and interpretation of data, draft of the work, development of intellectual content, writing of the manuscript, final approval of the version. JS: Draft of the work, developmente of intellectual content, final approval of the version. MP: Analysis and interpretation of data, draft of the work, development of intellectual content, final approval of the version. TF: Draft of the work, development of intellectual content, final approval of the version. ES: Analysis of data, development of intellectual content, final approval of the version. LS: Interpretation of data, development of intellectual content, writing of the manuscript, final approval of the version. SdS: Interpretation of data, development of intellectual content, writing of the manuscript and final approval of the version. GY: Analysis and interpretation of data, draft of the work, development of intellectual content, writing of the manuscript, final approval of the version.

### Conflict of interest statement

SdS was employed by company AES Tietê. The other authors declare that the research was conducted in the absence of any commercial or financial relationships that could be construed as a potential conflict of interest. The authors declare that this study received funding from AES Tietê (Research & Development Project #0064-1052/2014). The funder was not involved in the study design or collection, analysis, or interpretation of the data. The reviewer DH declared a shared affiliation, with no collaboration, with one of the authors MP to the handling Editor.

## References

[B1] Adamov N. S. M. Nascimento N. F. Maciel C. S. E. Santos M. P. Senhorini J. A. Calado L. L. (2016). Triploid induction in the *Yellowtail tetra, Astyanax altiparanae*, using temperature shock: tools for conservation and aquaculture. J. World Aquac. Soc. [Epub ahead of print]. 10.1111/jwas.12390

[B2] Allen S. K. (1983). Flow cytometry: assaying experimental polyploid fish and shellfish. Aquaculture 33, 317–328. 10.1016/0044-8486(83)90412-X

[B3] Allen S. K. Stanley J. G. (1978). Reproductive sterility in polyploid brook trout, salvelinus fontinalis. Trans. Am. Fish. Soc. 107, 473–478. 10.1577/1548-8659(1978)107<473:RSIPBT>2.0.CO;2

[B4] Allen S. K. Stanley J. G. (1979). Polyploid mosaics induced by cytochalasin b in landlocked atlantic salmon salmo salar. Trans. Am. Fish. Soc. 108, 462–466. 10.1577/1548-8659(1979)108<462:PMIBCB>2.0.CO;2

[B5] Allen S. K. Gagnon P. S. Hidu H. (1982). Induced triploidy in the soft-shell clam: cytogenetic and allozymic confirmation. J. Hered. 73, 421–428. 10.1093/oxfordjournals.jhered.a109691

[B6] Arumuganathan K. Earle E. D. (1991). Nuclear DNA content of some important plant species. Plant Mol. Biol. Rep. 9, 208–218. 10.1007/BF02672069

[B7] Brown B. L. Schultz S. L. W. White F. K. H. (2000). A convenient field method of tissue preservation for flow cytometric ploidy assessment of grass carp. Trans. Am. Fish. Soc. 129, 1354–1359. 10.1577/1548-8659(2000)129<1354:ACFMOT>2.0.CO;2

[B8] Burns E. R. Anson J. F. Hinson W. G. Pipkin J. L. Kleve M. G. Goetz R. C. (1986). Effect of fixation with formalin on flow cytometric measurement of DNA in nucleated blood cells. Aquaculture 55, 149–155. 10.1016/0044-8486(86)90070-0

[B9] Ciudad J. Cid E. Velasco A. Lara J. M. Aijón J. Orfao A. (2002). Flow cytometry measurement of the DNA contents of G0/G1 diploid cells from three different teleost fish species. Cytometry 48, 20–25. 10.1002/cyto.10100 12116377

[B10] de Siqueira-Silva D. H. Silva A. P. S. Ninhaus-Silveira A. Veríssimo-Silveira R. (2015). The effects of temperature and busulfan (Myleran) on the *Yellowtail tetra Astyanax altiparanae* (Pisces, Characiformes) spermatogenesis. Theriogenology 84, 1033–1042. 10.1016/j.theriogenology.2015.06.004 26164805

[B11] do Nascimento N. F. Pereira-Santos M. Piva L. H. Manzini B. Fujimoto T. Senhorini J. A. (2017). Growth, fatty acid composition, and reproductive parameters of diploid and triploid yellowtail tetra *Astyanax altiparanae*. Aquaculture 471, 163–171. 10.1016/j.aquaculture.2017.01.007

[B12] dos Santos M. P. Yasui G. S. Xavier P. L. P. Adamov N. S. M. Nascimento N. F. Fujimoto T.. (2016). Morphology of gametes, post-fertilization events and the effect of temperature on the embryonic development of *Astyanax altiparanae* (Teleostei, Characidae). Zygote 24, 1–13. 10.1017/S0967199416000101 27220819

[B13] Dpoolezel J. Binarova P. and S. Lucretti (1989). Analysis of nuclear DNA content in plant cells by flow cytometry. Biol. Plant. 31, 113–120. 10.1007/BF02907241

[B14] Galbraith D. W. Harkins K. R. Maddox J. M. Ayres N. M. Sharma D. P. Firoozabady E. (1983). Rapid flow cytometric analysis of the cell cycle in intact plant tissues. Science 220, 1049–1051. 10.1126/science.220.4601.1049 17754551

[B15] Kamiya E. Izumiyama S. Nishimura M. Mitchell J. G. Kogure K. (2007). Effects of fixation and storage on flow cytometric analysis of marine bacteria. J. Oceanogr. 63, 101–112. 10.1007/s10872-007-0008-7

[B16] Lampert K. P. Lamatsch D. K. Fischer P. Schartl M. (2008). A tetraploid amazon molly, *Poecilia formosa*. J. Hered. 99, 223–226. 10.1093/jhered/esm102 18209110

[B17] Loureiro J. Rodriguez E. Dolezel J. Santos C. (2007). Two new nuclear isolation buffers for plant DNA flow cytometry: a test with 37 species. Ann. Bot. 100, 875–888. 10.1093/aob/mcm152 17684025 PMC2749623

[B18] Mair G. C. (1993). Chromosome-set manipulation in tilapia - techniques, problems and prospects. Aquaculture 111, 227–244. 10.1016/0044-8486(93)90040-6

[B19] Marie D. Brown S. C. (1993). A cytometric exercise in plant DNA histograms, with 2C values for 70 species. Biol. Cell 78, 41–51. 10.1016/0248-4900(93)90113-S 8220226

[B20] Martinez E. R. M. Alves A. L. Silveira S. M. Foresti F. Oliveira C. (2012). Cytogenetic analysis in the incertae sedis species *Astyanax altiparanae* Garutti and Britzki, 2000 and *Hyphessobrycon eques* Steindachner, 1882 (Characiformes, Characidae) from the upper paraná river basin. Comp. Cytogenet. 6, 41–51. 10.3897/compcytogen.v6i1.1873 24260651 PMC3833770

[B21] Medina-Robles V. M. Velasco-Santamaría Y. M. Cruz-Casallas P. E. (2005). Aspectos generales de la crioconservación espermática en peces teleósteos. Rev. Col. Cienc. Pec. 18, 34–48. Available online at: http://www.scielo.org.co/pdf/rccp/v18n1/v18n1a05.pdf

[B22] Pfosser M. Amon A. Lelley T. Heberle-Bors E. (1995). Evaluation of sensitivity of flow cytometry in detecting aneuploidy in wheat using disomic and ditelosomic wheat-rye addition lines. Cytometry 21, 387–393. 10.1002/cyto.990210412 8608738

[B23] Piferrer F. Beaumont A. Falguière J. C. Flajšhans M. Haffray P. Colombo L. (2009). Polyploid fish and shellfish: production, biology and applications to aquaculture for performance improvement and genetic containment. Aquaculture 293, 125–156. 10.1016/j.aquaculture.2009.04.036

[B24] Prioli S. M. A. P. Prioli A. J. Júlio F. J. Pavanelli C. S. Oliveira A. V. Carrer H. (2002). Identification of *Astyanax altiparanae* (Teleostei, Characidae) in the Iguaçu River, Brazil, based on mitochondrial, DNA and RAPD markers. Genet. Mol. Biol. 25, 421–430. 10.1590/S1415-47572002000400011

[B25] Suganuma A. Morioka H. (1979). Morphological changes in membrane systems of staphylococci after different fixation procedures. J. Elect. Micros. 28, 29–35. 572856

[B26] Troussellier M. Courties C. Vaquer A. (1993). Recent applications of flow-cytometry in aquatic microbial ecology. Biol. Cell 78, 111–121. 10.1016/0248-4900(93)90121-T 8220221

[B27] Yamaha E. Saito T. Kazeto R. G. Arai K. (2007). Developmental biotechnology for aquaculture, with special reference to surrogate production in teleost fishes. J. Sea Res. 58, 8–22. 10.1016/j.seares.2007.02.003

[B28] Zhou X. Y. Li M. Y. Abbas K. Gao Z. X. Wang W. M. (2008). Comparison of ploidy level screening methods in chinese dojo loach (*Misgurnus anguillicaudatus*). J. Appl. Ichthyol. 24, 664–669. 10.1111/j.1439-0426.2008.01116.x

[B29] Zhu D. Song W. Yang K. Cao X. Gul Y. Wang W. (2012). Flow cytometric determination of genome size for eight commercially important fish species in China. In Vitro Cell. Dev. Biol. 48, 507–517. 10.1007/s11626-012-9543-7 22956044

